# Analysis of *Corynebacterium diphtheriae* macrophage interaction: Dispensability of corynomycolic acids for inhibition of phagolysosome maturation and identification of a new gene involved in synthesis of the corynomycolic acid layer

**DOI:** 10.1371/journal.pone.0180105

**Published:** 2017-07-07

**Authors:** Lisa Ott, Elena Hacker, Timo Kunert, Ian Karrington, Philipp Etschel, Roland Lang, Veit Wiesmann, Thomas Wittenberg, Albel Singh, Cristian Varela, Apoorva Bhatt, Vartul Sangal, Andreas Burkovski

**Affiliations:** 1 Mikrobiologie, Friedrich-Alexander-Universität Erlangen-Nürnberg, Erlangen, Germany; 2 Mikrobiologisches Institut, Universitätsklinikum Erlangen, Friedrich-Alexander-Universität Erlangen-Nürnberg, Erlangen, Germany; 3 Fraunhofer Institut für Integrierte Schaltungen (IIS), Erlangen, Germany; 4 Institute of Microbiology and Infection and School of Biosciences, University of Birmingham, United Kingdom; 5 Faculty of Health and Life Sciences, Northumbria University, Newcastle upon Tyne, United Kingdom; Centre National de la Recherche Scientifique, FRANCE

## Abstract

*Corynebacterium diphtheriae* is the causative agent of diphtheria, a toxin mediated disease of upper respiratory tract, which can be fatal. As a member of the CMNR group, *C*. *diphtheriae* is closely related to members of the genera *Mycobacterium*, *Nocardia* and *Rhodococcus*. Almost all members of these genera comprise an outer membrane layer of mycolic acids, which is assumed to influence host-pathogen interactions. In this study, three different *C*. *diphtheriae* strains were investigated in respect to their interaction with phagocytic murine and human cells and the invertebrate infection model *Caenorhabditis elegans*. Our results indicate that *C*. *diphtheriae* is able to delay phagolysosome maturation after internalization in murine and human cell lines. This effect is independent of the presence of mycolic acids, as one of the strains lacked corynomycolates. In addition, analyses of NF-κB induction revealed a mycolate-independent mechanism and hint to detrimental effects of the different strains tested on the phagocytic cells. Bioinformatics analyses carried out to elucidate the reason for the lack of mycolates in one of the strains led to the identification of a new gene involved in mycomembrane formation in *C*. *diphtheriae*.

## Introduction

The genus *Corynebacterium* belongs to the class of *Actinobacteria* and comprises a collection of morphologically similar, irregular- or club-shaped non-sporulating (micro-)aerobic microorganisms. To date, 90 species were taxonomically classified [1,2]. More than half of these, i.e. 52 species, are occasional or rare causes of infections, while only a few are evoking severe diseases. The most prominent member of the latter group is *Corynebacterium diphtheriae*, which is also the type species of the genus. *C*. *diphtheriae* is the etiological agent of respiratory diphtheria, which is restricted with about 5,000 annual reports mainly to developing countries today, but nevertheless caused a severe outbreak with more than 157,000 cases in the states of the former Soviet Union in 1990 to 1998 [3]. In addition, a number of outbreaks have recently been reported from different countries [4].

Together with the genera *Mycobacterium*, *Nocardia* and *Rhodococcus*, the genus *Corynebacterium* forms the CMNR group within the high G + C Gram-positive bacteria. Almost all members of the CMNR group are characterized by a mycolic acid layer, the mycomembrane, which covers the peptidoglycan and is in many aspects functionally equivalent to outer membrane of Gram-negative bacteria [5,6]. Mycolic acids are α-alkylated β-hydroxylated fatty acids with a short α-alkyl and a meromycolate side chain, which can comprise between 12–18 carbon atoms in case of corynebacteria [7] and 67–75 carbon atoms in mycobacteria. On the one hand, the mycolic acids are covalently linked to the arabinogalactan-peptidoglycan meshwork of the cell wall on the other hand to distinct sugars forming glycolipids, which are located in the outer leaflet of the mycomembrane. A prominent member of these glycolipids is trehalose dimycolate (TDM). TDM is involved in host-pathogen interaction by *Mycobacterium tuberculosis* and *Rhodococcus equi* [8–10] and consequently of high interest in respect to pathogenicity of bacteria.

While *M*. *tuberculosis* mycolic acid synthesis, and the role of trehalose dimycolates in virulence is well studied [11–15], only very limited information is available for corynebacterial mycolates. Investigations of *Corynebacterium pseudotuberculosis* indicated a lethal effect of mycomembrane lipids on caprine and murine macrophages. Lipid extracts of *C*. *pseudotuberculosis* had negative effects on glycolytic activity, membrane integrity and viability of cells [16]. Furthermore, recent investigations on *Corynebacterium ulcerans* indicated that the bacteria are able to delay phagolysosome maturation in macrophages [17], a process resembling the situation of *M*. *tuberculosis*-macrophage interaction. Since we are interested in interaction of *C*. *diphtheriae* with host cells [18–22], these studies prompted us to investigate the influence of mycolic acids on the interaction with macrophage-like cell lines. Three different *C*. *diphtheriae* strains were chosen: the non-toxigenic isolate DSM43988, the toxin-producer DSM43989 and the type strain DSM44123, which is also non-toxigenic. Strain DSM43989 is a PW8 strain used for toxoid production. In previous studies, DSM43989 showed low adhesion to and invasion into epithelial cells in comparison to other non-toxigenic isolates [21] and we were interested to elucidate the reason for this behavior. The number and types of surface pili are important for adhesion and invasion [19,23,24]; however, their presence has been verified by atomic force microscopy [19,23]. Recently, an influence of the mycolate profile on adherence properties of *M*. *tuberculosis* was shown [25] and also lineage-specific trends in mycolic acid repertoire were described [26]. Therefore, we tested whether a different glycolipid composition might be the reason for different adhesion patterns.

## Material and methods

### Bacterial strains and growth conditions

*C*. *diphtheriae* strains (Table 1) were grown at 37°C in Heart Infusion (HI) broth (Becton Dickinson, Sparks, MD, USA). For the determination of doubling times and recording of growth curves, bacteria from an overnight culture were inoculated to an OD_600_ of approx. 0.1 and growth was followed spectrophotometrically. Experiments were carried out at least in triplicates (biological replicates). For solid media either HI Agar, Brain Heart Infusion Agar (Oxoid, Basingstoke, UK) or Columbia Blood Agar plates containing 5% sheep blood were used (Oxoid, Basingstoke, UK).

**Table 1:** Bacterial strains, plasmids and cell lines used in this study.

*C. diphtheriae* strains	Genotype/Description	Reference/Source
DSM43988	Strain 48255, ATCC 11913, avirulent throat culture	DSMZ, Braunschweig, Germany
DSM43989	PW strain, strain 5159, ATCC 13812, producer of diphtheria toxin for toxoid production	[27], DSMZ, Braunschweig, Germany
DSM44123	Type strain, C7^s^, ATCC 27010, CIP 100721, NCTC 11397	DSMZ, Braunschweig, Germany
***C*. *glutamicum* strains**		
ATCC 13032	Type strain	[28]
*Cg-Δpks*	*C*. *glutamicum pks13* deletion strain	[29]
**Plasmids**		
pEPR1-p45gfp	p*45*, *gfpuv*, Km^R^, *rep*, *per*, T1, T2	[30]
pK18*mob*	Insertion vector, Km^R^, *ori* pUC, *mob*, 3793 bp	[31]
pK18*mob*-B178_*03333‘-2*	pK18*mob* with 500 bp insertion of gene *B178_03333*	Kindly provided by C. Bolz, Erlangen
pZ8-1	Expression vector, p*tac*, Km^R^, *ori* pUC, *ori C*. *glutamicum*, 7072 bp	Degussa AG, Halle, Germany
pZ8-1_*B178_03333*	pZ8-1 carrying the complete *B178_03333* gene	This work
**Cell lines**		
J774E	Mannose receptor-expressing clone of the J774 mouse macrophage cell line	[32]
THP-1	Human leukemic monocytic cells	[33]
THP1-Blue NFκB	THP-1 cells with stable integrated NFκB inducible SEAP reporter construct	Invivogen

For generation of GFP expressing strains used in fluorescence microscopy studies, electro-competent *C*. *diphtheriae* were transformed with the plasmid pEPR1-p45gfp and positive clones were selected on HI agar with kanamycin (25 μg ml^-1^ final concentration).

### Determination of minimal inhibitory concentrations of antibiotics

For the determination of minimal inhibitory concentrations (MICs) of antibiotics, MIC Evaluator strips were used as recommended by the supplier (Oxoid, Basingstoke, UK). In short, 100 μl of *C*. *diphtheriae* exponential phase cultures (OD_600_ approx. 0.3) were plated on HI agar in 90 mm petri-dishes. Strips were placed on the agar surface followed by an overnight incubation at 37°C. Based on the growth inhibition observed, MICs were directly read-out according to the manufacturer’s protocol.

### Lipid extraction, thin-layer chromatography and staining

*C*. *diphtheriae* strains were grown in shaking flasks to the exponential (OD_600_ approx. 0.5), harvested, washed with PBS and used for lipid extraction. Protocols for lipid and mycolic acid extraction were the same as used for *C*. *glutamicum* [29,34]. Extracts of methyl esters of corynomycolic acids (CMAMES) were separated by thin layer chromatography on a silica gel plate using petroleum ether:acetone (95:5 v/v). CMAMES and other fatty acyl species were visualized by staining with molybdophosphoric acid and subsequent charring. Total lipid extracts were separated using chloroform:methanol:water (60:16:2 v/v/v) and visualized by staining with alpha naphtol and subsequent charring.

### Nematode killing assay

*C*. *elegans* N2 was maintained on *E*. *coli* OP50 for six to seven days until the worms became starved, as described above [35]. Infection of L4 stage larval worms was carried out with 20 μl of each bacterial strain (from an overnight culture) on NGM plates at 21°C for 24 h. Worms were assessed each day following infection, and the dead nematodes were counted and removed every 24 h. For each strain, approximately 20 nematodes were used and the assays were performed in triplicates.

### Replication assay

Murine macrophage-like J774E cells and human monocytic THP-1 cells were cultured in 10% FCS supplemented RPMI medium 1640 (containing 100 U penicillin ml^-1^ and 100 mg streptomycin ml^-1^) at 37°C with 5% CO_2_ in a humidified incubator. For replication assays, THP-1 cells were seeded in 24-well plates (Nunc) at a density of 2 x 10^5^ and differentiated by addition of 10 ng ml^-1^ phorbol 12-myristate 13-acetate (PMA) 24 h prior to infection. J774E cells were seeded at a density of 1 x 10^5^ in 24 well plates 24 h prior to infection. Overnight cultures of *C*. *diphtheriae* grown in HI were re-inoculated to an OD_600_ of 0.1 in fresh medium and grown to an OD_600_ of 0.4 to 0.6. An inoculum with an MOI of 1 or 10 was prepared in RPMI 1640 without antibiotics and 500 μl per well were used to infect the cells. The plates were centrifuged for 5 min at 350 x *g* to synchronize infection and incubated for 30 min (37°C, 5% CO_2_, 90% humidity) to allow phagocytosis of bacteria. Subsequently, the supernatant containing non-engulfed bacteria was aspirated, cells were washed once with PBS and remaining extracellular bacteria were killed by addition of 100 μg ml^-1^ gentamicin in cell culture medium. After 2 h, cells were either lysed and intracellular bacteria were recovered or further incubated with medium containing 10 μg ml^-1^ gentamicin for analysis at later time points (8 h and 20 h). To recover intracellular bacteria, the medium was aspirated and cells were lysed by adding 500 μl of 0.1% Triton-X100 in PBS. Serial dilutions of lysate and inoculum were plated on blood agar plates using an Eddy Jet Version 1.22 (IUL Instruments). After incubation at 37°C for two days, the number of colony forming units (CFU) was determined. The ratio of bacteria used for infection (number of colonies on inoculum plates) and bacteria in the lysate (number of colonies on the lysate plates) multiplied with 100 gave the percentage of viable intracellular bacteria at different time points. When the survival of intracellular bacteria in THP-1 cells was analyzed over the time, the number of CFU at 2 h was set to 100% and later time points were calculated based on this value. The assay was performed in three biological replicates each performed in triplicates and the mean values were calculated with standard deviations.

### Fluorescence microscopy

For qualitative analysis of intracellular CFU, J774E and THP-1 cells were infected with GFP-expressing bacteria and analyzed by fluorescence microscopy. Cells were seeded one day prior to infection in a density of 1 x 10^5^ cells on sterile coverslips in 24-well plates. Overnight cultures of *C*. *diphtheriae* strains transformed with plasmids encoding *gfp* cultivated in HI medium containing kanamycin were re-inoculated to an OD_600_ of 0.1 in fresh medium, harvested at the beginning of the exponential growth phase and used to infect macrophages as described above. After different time points, the medium was aspirated and cells were fixed by addition of 500 μl 4% paraformaldehyde in PBS and incubated for 20 min at 37°C. The cells were stored in PBS at 4°C until tested for internalization by staining. For subsequent analysis by microscopy, coverslips were incubated with 30 μl of Alexa Fluor 647 Phalloidin diluted 1:200 in Image-iT FX Signal Enhancer (Molecular Probes, Life Technologies) for 45 min in the dark to stain the cytoskeleton of THP-1 cells. After washing twice with PBS, the coverslips were dried and embedded on glass slides in ProLong Gold antifade mountant with DAPI (Molecular Probes, Life Technologies) and samples were stored in the dark at 4°C. Micrographs were taken with the confocal laser scanning microscope Leica SP5 CLSM-1P (Leica Microsystems) and analyzed with the LAS software suite.

### Staining of acidic compartments in infected macrophages

To analyze if *C*. *diphtheriae* co-localizes with acidic compartments, J774E and THP-1 cells we were treated with 200 nM LysoTracker Red DND-99 (Molecular Probes, Life Technologies), a red fluorescent dye that stains acidic compartments in live cells, 2 h before infection. Then, cells were infected and further treated as described for fluorescence microscopy above.

### Automated analysis of fluorescence microscopic pictures

The analysis of bacteria on fluorescence images poses a demanding challenge, as the bacteria tend to stick together and form clusters. To avoid putative pitfalls due to problems with the segmentation of single bacteria, data were analyzed in two ways [17]. In the first approach, the area of all bacteria was analyzed on pixel level. For this purpose, an adaptive threshold based on k-means clustering was applied to the images of the GFP channel to determine pixels, which belong to bacteria. For each image the amount of co-localizing pixels were determined by measuring the intensity values of corresponding pixels in the image of the LysoTracker Red DND-99 channel. These intensities were compared to the threshold intensity value. Above the empirically determined intensity threshold value the pixel were regarded as co-localized. The second method was based on the evaluation of images at bacterial cell level. For this purpose, pixels belonging to bacteria were grouped into regions. To prevent the analysis of cell clusters, these regions were filtered according to a statistical shape model to analyze exclusively single bacteria, which had to reach a minimum average intensity in the LysoTracker Red DND-99 channel to be positive for co-localization with acidic compartments.

### NF-κB reporter assay

THP1-Blue NF-κB cells (InvivoGen) carrying a stable integrated NF-κB-inducible secreted embryonic alkaline phosphatase (SEAP) reporter construct were used to analyze NF-κB induction. For this purpose, *C*. *diphtheriae* strains were inoculated to an OD_600_ of 0.1 in HI medium and grown until an OD_600_ of 0.4–0.6 was reached. An inoculum with OD_600_ of 1 in 1000 μl PBS was prepared and 20 μl of this inoculum or of 10^−1^ and 10^−2^ dilutions were mixed with 180 μl of a suspension with 5 x 10^5^ THP1-Blue NF-κB cells in cell culture medium resulting in MOIs of 100, 10 and 1. UV-killed bacteria in the same concentrations were used as control. After incubation for 20 h, the 96-well plates were centrifuged (350 x *g*, 5 min) and 20 μl of the cell free supernatant was mixed with pre-warmed SEAP detection reagent QUANTI-Blue (InvivoGen). After further incubation at cell culture conditions for 3 h, the levels of NF-κB-induced SEAP resulting in a color change from pink to blue was measured in a microplate reader (TECAN Infinite 200 PRO) at 620 nm.

### Determination of cytokine excretion

For determination of cytokine activation by the different *C*. *diphtheriae* strains, supernatants of infected THP-1 cells were collected at different time points and stored at -20°C. IL-6 and G-CSF concentrations were measured using the DuoSet ELISA Kits according to the manufacturer’s recommendations (R&D systems). Briefly, the ELISA plates were coated over night with a capture antibody at room temperature, washed three times with 0.05% Tween20 in PBS, blocked for 1 h at room temperature with 1% BSA in PBS and washed again three times. Subsequently, 100 μl samples of supernatant of infected cells or standard dilutions were added and the plates were incubated for 2 h, washed again three times and further incubated for 2 h at room temperature. After another washing step, a streptavidin-HRP solution was added and the plates were stored for 20 min under light exclusion, washed again and incubated for another 20 min in the dark with substrate solution. To stop color development, 2 N H_2_SO_4_ was added to the wells and the optical density was determined using a microplate reader (TECAN Infinite 200 PRO) set to 450 nm with wavelength correction at 550 nm.

### Genome sequencing and analysis

Genomic DNA of strains DSM43989 and DSM44123 were extracted from 1.5 ml cultures grown overnight at 37°C in BHI broth (Oxoid) using UltraClean Microbial DNA Isolation Kit (MoBio) and were sequenced on an Illumina MiSeq instrument, according to the manufacturer’s instructions. The reads were assembled into contigs using SPADes 3.6.2 with a value of k = 127 [36]. The draft assemblies were submitted to the PGAP pipeline for annotation [37]. The sequence types (STs) of these strains were identified by using MLST v 1.8 [38].

The protein sequence of genes reported to be involved in mycolic acid biosynthesis [39,40] and trehalose biosynthesis [41] were searched using BLAST [42] in the genomes of strains DSM43989, DSM44123 and DSM43988 (Accession No. AUZN00000000; [2]). Additional genes with potential carboxylase, esterase/hydrolase, phosphopentethenyl transferase, acyl-CoA synthetase and enoyl-CoA hydratase activities were identified in the genome of strain NCTC 13129 (Accession No. BX248353; [43]) using UniProt database (http://www.uniprot.org/) and were also searched in DSM43989, DSM44123 and DSM43988 genomes.

### Construction of DIP0789 homolog B178_03333 overexpression plasmid

Standard techniques were used for plasmid isolation, transformation and cloning [44]. For cloning of the overexpression vector the gene *B178_03333* was amplified by PCR using chromosomal DNA of strain DSM43988 as template and the following primers: 5´-CGCGGGATCCGTGGCGCAGGTAGAGGTGCG-3´ and 5´-CGCGGTCGACGAATCAGCGACCAGTAAAC-3´. Using the BamHI and SalI sites introduced *via* the PCR primers the DNA fragment was ligated to BamHI/SalI-restricted and dephosphorylated pZ8-1 DNA. The resulting overexpression plasmid

pZ8-1_*B178_03333* was amplified *in E*. *coli* DH5αMCR. One microgram of unmethylated plasmid isolated from this *E*. *coli* strain was used to transform *C*. *diphtheriae* DSM43989 using a GenePulser II (Bio-Rad, Munich Germany). Electroporated cells were added to 1 ml of HI broth and incubated for 2 h at 37°C. An appropriate volume of culture was plated on medium containing kanamycin.

### Construction of insertion plasmid pK18*mob*_*B178*_*03333*´

For cloning of the insertion vector a 500 bp fragment of the gene *B178_03333* was amplified by PCR using chromosomal DNA of strain DSM43988 as template and the following primers:

5´-CGCGCCCGGGGTGCGATGATATTACATCTG-3´and 5´-GCGCCCGGGCCATTCCAGCAATACGATG-3´. Using the XmaI site introduced *via* the PCR primers the DNA fragment was ligated to XmaI-restricted and dephosphorylated pK18*mob* DNA. The resulting vector pk18*mob*_*B178_03333´* was then amplified *in E*. *coli* DH5αMCR. One microgram of unmethylated plasmid isolated from this *E*. *coli* strain was used to transform *C*. *diphtheriae* DSM43988 using a GenePulser II (Bio-Rad, Munich Germany). Electroporated cells were added to 1 ml of HI broth and incubated for 2 h at 37°C. An appropriate volume of culture was plated on medium containing kanamycin.

## Results

### Growth behavior of *C*. *diphtheriae* isolates

**Fig 1:**
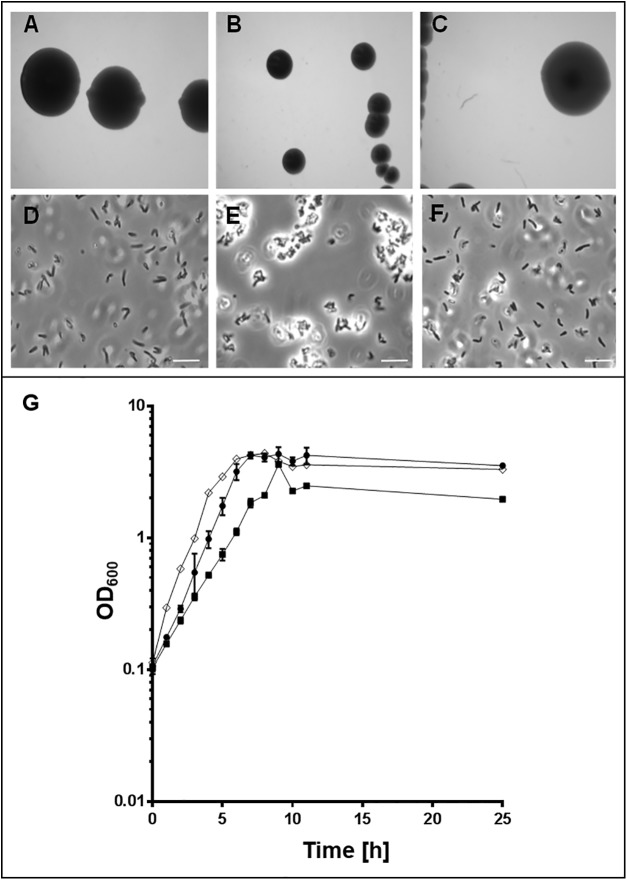
Growth properties of *C*. *diphtheriae* isolates. Colonies of *C*. *diphtheriae* strains DSM43988 (A), DSM43989 (B) and DSM44123 (C) grown on HI medium agar plates. Microscopic images of *C*. *diphtheriae* strains DSM43988 (D), DSM43989 (E) and DSM44123 (F) (scale bar: 10 μm). (G) Growth curve of the corresponding strains. DSM43988, filled circle; DSM43989, filled square; DSM44123, open rhombus. Experiments were carried out in triplicates (biological replicates) and standard deviations are shown.

When *C*. *diphtheriae* strain DSM43988, DSM43989 and DSM44123 were streaked-out on BHI, Columbia Blood agar or HI agar plates, colonies of DSM43989 appeared to be more whitish and streak-outs seemed to be more confluent. Furthermore, biomass formation was impaired compared to the other strains leading to a significantly diminished colony size of strain DSM43989 (Fig 1A–1C). In accordance with this observation, analysis of growth in HI liquid medium revealed a doubling time of 106 ± 2 min for DSM43989 compared to 72 ± 4 min for DSM43988 and 64 ± 6 min for DSM44123 (data are mean values of at least three independent biological replicates ± standard deviation; see also Fig 1G). Additionally, microscopic inspection of HI-grown cultures showed a tendency of aggregation in case of DSM43989, indicating altered surface properties (Fig 1D–1F).

### Corynomycolic acid profiles of *C*. *diphtheriae* strains

**Fig 2:**
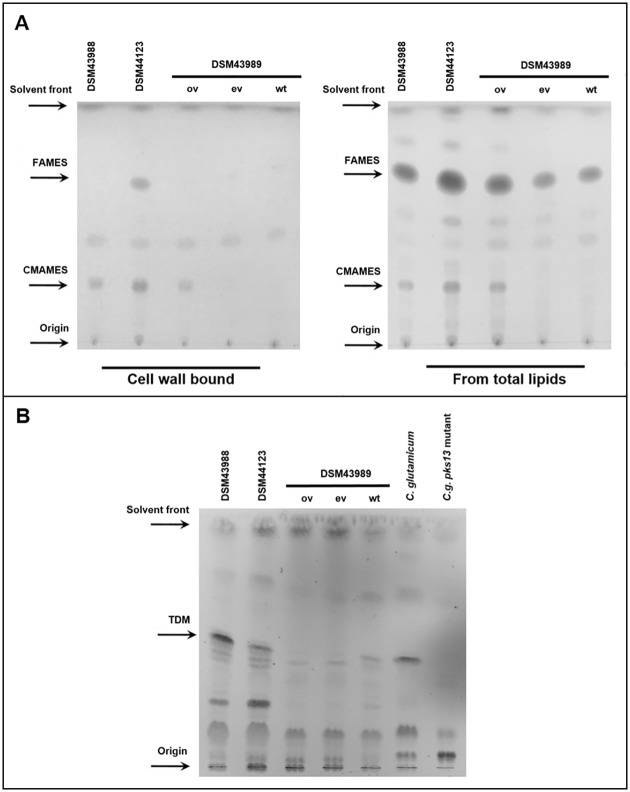
TLC analysis of cell wall extracts from different *C*. *diphtheriae* strains. (A) Methyl esters of corynomycolic acids (CMAMES) extracted using petroleum ether:acetone (95:5 v/v) from either total lipids (representing trehalose-bound corynomycolates) or delipidated cells (representing cell wall-bound corynomycolates) were separated by TLC and visualized by staining with phosphomolybdic acid (MPA) and charring. (B) TLC analysis of total lipid extracts from *Corynebacterium* strains using chloroform:methanol:water (60:16:2 v/v/v). Fractions are visualised by charring with alpha naphtol. TDM: trehalose dimycolate; *C*. *glutamicum* wild type was used as positive and the *pks13* mutant *Cg-Δpks* as negative control. Results for DSM43989 are shown for untransformed strain (wt), transformed with the empty vector pZ8-1 (ev) and complemented by transformation with pZ8-1_*B178_03333* (ov).

Clumpy and slow growth are often a result of loss of corynomyclic acid biosynthesis [45–47]. We thus analysed the DSM43989 strain for the presence of corynomycolic acids. Methyl esters of corynomycolic acids (CMAMES) extracted from either total lipids (representing trehalose-bound corynomycolates) or delipidated cells (representing cell wall-bound corynomycolates) were separated by TLC and visualized by staining with phosphomolybdic acid (MPA) and charring. A drastic phenotype was observed for DSM43989 (Fig 2A): no free mycolates or mycolates attached to the cell wall were detectable using thin-layer chromatography, indicating that the strain either lost the ability to synthesis mycolates or produces a precursor that is labile to the extraction process. This result was confirmed by thin-layer chromatography and direct visualization of trehalosyl dimycolates (TDM) (Fig 2B). As in case of a *C*. *glutamicum pks13* mutant, no TDM was detectable in DSM43989.

### Resistance to antibiotics

For mutant strains of *M*. *tuberculosis* with altered mycolic acid composition, changes in the resistance to antibiotics were reported previously [12,48]. In this study, a strong impact of the mycolic acid layer on antibiotics resistance of *C*. *diphtheriae* was observed. Determination of minimal inhibitory concentrations revealed a more than ten-fold increased sensitivity of strain mycolic acid-free strain DSM43989 towards β-lactam antibiotics (ampicillin, amoxicillin, imipenem and penicillin G) compared to the mycolic acid-containing isolates. An about four- to fivefold increased sensitivity of DSM43989 against tetracycline and the aminoglycoside gentamicin was observed. The effects for the glycopeptide vancomycin and the oxazolidione linezolid were weaker showing a twofold increase in sensitivity and no difference was observed for sensitivity against the macrolides clindamycin and erythromycin (Table 2).

**Table 2:** Resistance of *C*. *diphtheriae* strains against different antibiotics. Experiments were carried out in triplicates (independent biological replicates) and minimal inhibitory concentrations (given in μg ml^-1^) were determined after 16 h of incubation at 37°C.

Antibiotics	DSM43988	DSM43989	DSM44123
Amoxycillin	0.25/0.25/0.25	< 0.015/< 0.015/0.03	0.25/0.25/0.25
Ampicillin	0.25/0.25/0.5	< 0.015/< 0.015/< 0.015	0.25/0.25/0.25
Clindamycin	0.06/0.06/0.06	0.06/0.06/0.06	0.06/0.06/0.06
Erythromycin	0.015/0.015/0.015	0.015/0.015/<0.015	0.015/0.015/0.015
Gentamicin	0.25/0.25/0.25	0.06/0.06/0.06	0.25/0.25/0.25
Imipenem	0.015/0.03/0.03	0.002/< 0.002/0.002	0.06/0.03/0.03
Linezolid	1.0/1.0/0.5	0.25/0.5/0.5	0.5/0.5/1.0
Penicillin G	0.12/0.12/0.25	0.015/0.015/<0.015	0.25/0.25/0.25
Tetracycline	0.25/0.5/0.5	0.06/0.06/0.12	0.5/0.5/0.5
Vancomycin	0.5/1.0/1.0	0.5/0.5/0.25	1.0/1.0/1.0

### Colonization of the invertebrate infection model *C*. *elegans*

**Fig 3:**
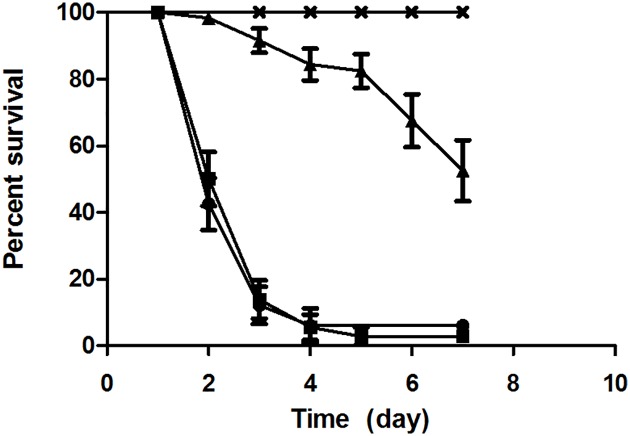
Nematode survival assay. (A) Infection of *C*. *elegans* N2 with *E*. *coli* OP50 (**x**), and *C*. *diphtheriae* strains DSM43988 (**■**), DSM43989 (▲) and DSM44123 (●). Data shown are the mean of three parallel experiments with 20 worms per plate repeated three times independently, error bars represent deviations from mean values.

**Fig 4:**
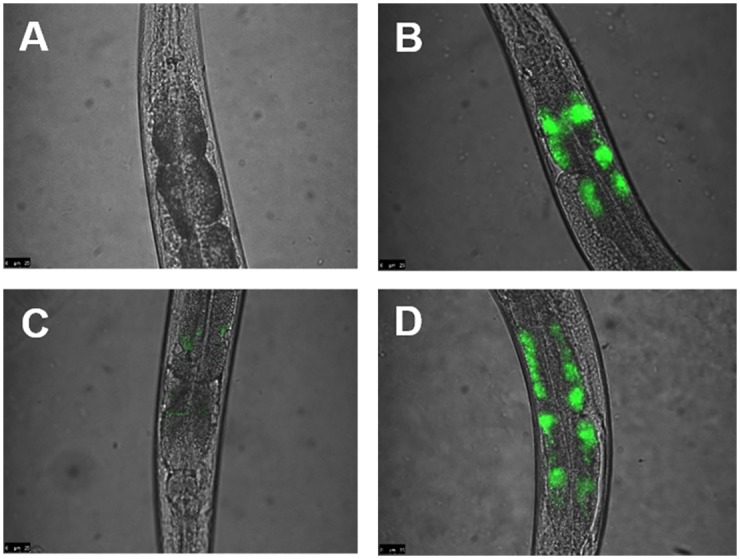
Fluorescence microscopy of *C*. *elegans* colonization. Nematodes infected with bacteria were mounted onto agar pads, paralyzed with 0.6% 2-phenoxy-2-propanol and photographed using a Leica DMI4000B. In each of three independent experiments, approximately 20 worms were infected. Representative results are shown. *C*. *elegans* fed with (A) *E*. *coli* OP50 pEPR1p45*gfp*, (B) *C*. *diphtheriae* DSM43988 pEPR1p45*gfp*, (C) *C*. *diphtheriae* DSM43989 pEPR1p45*gfp* and (D) *C*. *diphtheriae* DSM44123 pEPR1p45*gfp*.

In order to evaluate the consequences of mycolic acid deficiency in strain DSM43989, the colonization of *C*. *elegans*, an established infection model for corynebacteria [20,22,49,50], was tested. A nematode killing assays revealed a clear detrimental effect of DSM43989 on *C*. *elegans*, although the killing of worms by the mycolic acid-free strain was slower compared to DSM43988 and DSM44123 (Fig 3). This correlates with the slower growth of DSM43989 compared to DSM43988 and DSM44123. The observations made for the killing assay were in line with results of fluorescence microscopy analyses of *C*. *elegans* colonized by GFP-expressing bacteria (Fig 4). While the *E*. *coli* control showed no fluorescence (Fig 4A), the slow growing DSM43989 pEPR1-p45*gfp* showed a strongly reduced fluorescence signal (Fig 4C) compared to DSM43988 pEPR1-p45*gfp* and DSM44123 pEPR1-p45*gfp* (Fig 4B and 4D),

### Effect on survival in macrophage cell lines

As a more complex system, the different *C*. *diphtheriae* strains were analyzed in respect to their interaction with murine and human macrophage-like cells. While murine cell lines exhibit no diphtheria toxin receptor, human cells are susceptible to diphtheria toxin and consequently, it is possible to distinguish between the influence of mycolic acids and diphtheria toxin with the combination of macrophage cell lines used here.

**Fig 5:**
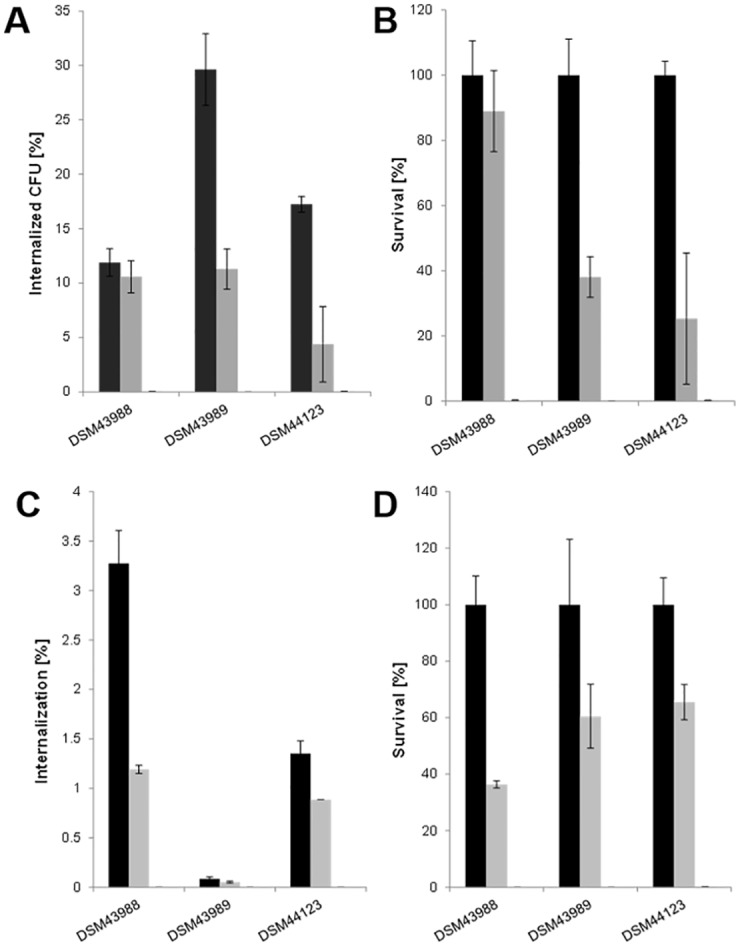
Replication of *C*. *diphtheriae* in macrophages. Uptake and survival of strains was tested in murine J774E (A, B) and human THP-1 (C, D) cells. The percentage of CFU in respect to the applied inoculum (A, C) and relative to the value 2 hours after infection set to 100% (B, D) is shown.

Murine J774E cells readily took up the bacteria without further priming and colony forming units were detectable after cell lysis 2, 8 and, to a minor extend also 20 hours after infection. Interestingly, DSM43989 showed the highest number of colony forming units of all strains after 2 hours (Fig 5A). For a better comparison of the kinetics of survival in macrophages murine, the 2 hour values were set to 100% and survival rates were calculated. In this case, DSM43988 was highly resistant to macrophage action with about 90% of the internalized bacteria being still viable after 8 hours. DSM43989 and DSM44123 showed a very similar behavior in respect to macrophage resistance with about 40 and 30% survival rate after 8 hours, respectively (Fig 5B).

When human THP1 cells were used, compared to the murine macrophages, a ten-fold decrease was observed in internalization rates. In this set-up DSM43989 showed very low internalization values (Fig 5C), while the kinetics of survival was better than in the murine system. Since tox^+^ strain DSM43989 and tox^-^ strain DSM44123 showed an almost identical survival rate (Fig 5D), it can be concluded that the diphtheria toxin had no dramatic influence in the time range tested. This observation is in accordance with previous observations made for *C*. *diphtheriae*-infected epithelial cells [19].

### Delay of phagolysosome maturation by *C*. *diphtheriae*

A hallmark of macrophage function after phagocytosis is the formation of phagolysosomes by fusion of phagosomes and acidic lysosomes in order to destroy the phagocytosed pathogens. It is well known for *M*. *tuberculosis* and *Rhodococcus equi* that mycolic acids influence phagosome maturation. Therefore, formation of acidic compartments within J774E and THP-1 macrophages was monitored using a LysoTracker dye. In parallel, Alexa Fluor 647 Phalloidin and DAPI staining visualized cytoskeleton and nuclei, respectively, and *Corynebacterium* strains were labeled by GFP.

**Fig 6:**
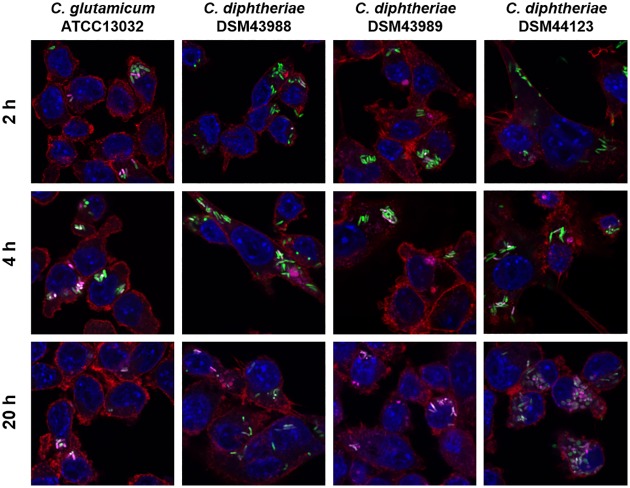
Labeling and tracking of acidic organelles in macrophages cells infected with *C*. *diphtheriae*. J774E cells were incubated with LysoTracker Red DND-99 for 120 min before cells were infected with *C*. *glutamicum* ATCC 13032 pEPR1p45*gfp* and *C*. *diphtheriae* strains DSM43988 pEPR1p45*gfp*, DSM43989 pEPR1p45*gfp* and DSM44123 pEPR1p45*gfp* at an MOI of 10 for 30 min. Extracellular bacteria were killed by the addition of gentamicin and after 2, 4 and 20 h, cells were fixed. Nuclei were stained with DAPI, the cytoskeleton with Alexa Fluor 647 Phalloidin and micrographs were taken using the confocal laser-scanning microscope Leica SP5 II and analysed with the LAS software suite. Non-pathogenic *C*. *glutamicum* immediately co-localize with acidic compartments (2 h) whereas *C*. *diphtheriae* strains only show co-localization after longer incubation time (4 h to 20 h). Representative pictures are shown. Scale bars: 10 μm.

When J774E cells (Fig 6) were infected, in case of *C*. *glutamicum* ATCC 13032 pEPR1p45*gfp*, which was used as control, the majority of bacteria was co-localized with acidic compartments already after 2 h with almost 100% co-localization after 4 h. This situation differed significantly in case of the different *C*. *diphtheriae* isolates as a very low rate of co-localization was detected after 2 h. After 4 hours, only about half of the bacteria were co-localizing with lysosomes, and only after 20 hours, the majority of GFP-labeled bacteria were co-localizing with acidic compartments.

**Fig 7:**
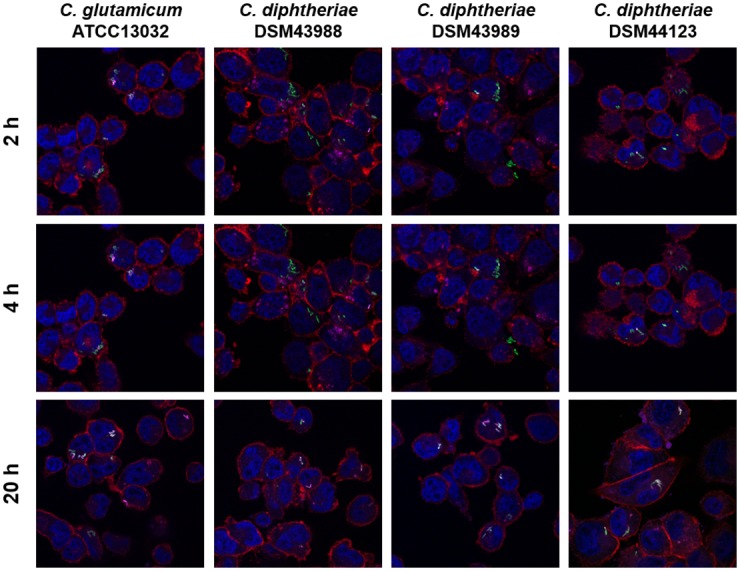
Labeling and tracking of acidic organelles in macrophages cells infected with *C*. *diphtheriae*. THP-1 cells were incubated with LysoTracker Red DND-99 for 120 min before cells were infected with *C*. *glutamicum* ATCC 13032 pEPR1p45*gfp* and *C*. *diphtheriae* strains DSM43988 pEPR1p45*gfp*, DSM43989 pEPR1p45*gfp* and DSM44123 pEPR1p45*gfp* at an MOI of 10 for 30 min. Extracellular bacteria were killed by the addition of gentamicin and after 2, 4 and 20 h, cells were fixed. Nuclei were stained with DAPI, the cytoskeleton with Alexa Fluor 647 Phalloidin and micrographs were taken using the confocal laser-scanning microscope Leica SP5 II and analysed with the LAS software suite. Non-pathogenic *C*. *glutamicum* immediately co-localize with acidic compartments (2 h) whereas *C*. *diphtheriae* strains only show co-localization after longer incubation time (4 h to 20 h). Representative pictures are shown. Scale bars: 10 μm.

A similar picture was observed with THP-1 cells (Fig 7). Again, co-localization with *C*. *glutamicum* was fast, while co-localization with *C*. *diphtheriae* strains DSM43988 pEPR1p45*gfp*, DSM43989 pEPR1p45*gfp* and DSM44123 pEPR1p45*gfp* was delayed. The data indicate a delay of phagolysosome formation in murine and human macrophage cell lines by *C*. *diphtheriae* independent of the presence or absence of mycolic acids.

**Fig 8:**
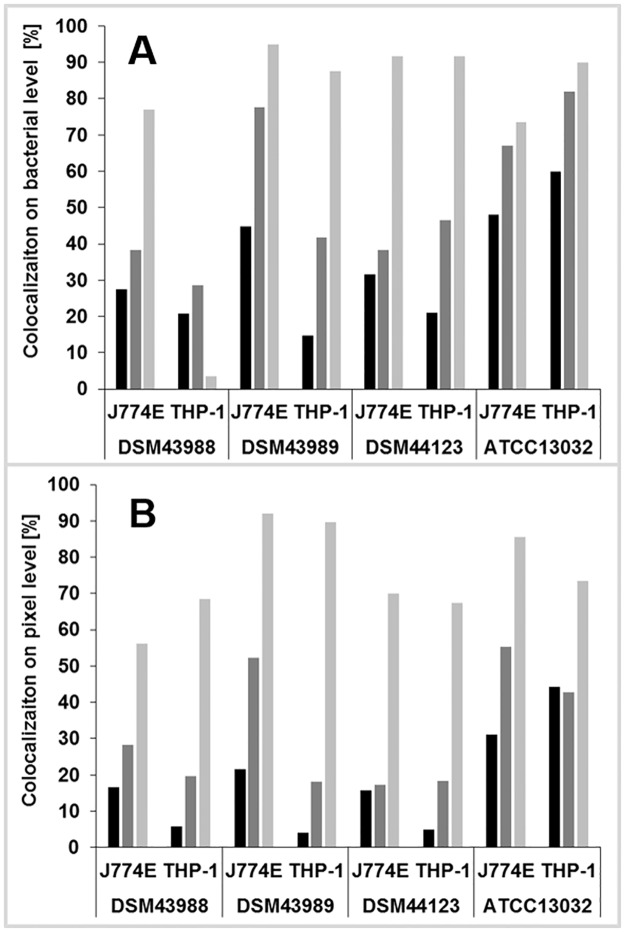
Automated analysis of co-localization of bacteria with acidic compartments. At least 8 fluorescence microscopy images were analyzed for each data set as described in the Materials and Methods section. (A) Co-localization of corynebacteria with acidic compartments at bacterial level and (B) co-localization at pixel level in murine J774E and human THP-1 macrophage-like cell lines.

For an unbiased and more quantitative approach, fluorescence microscopy images were automatically evaluated either at the level of bacterial cells or at pixel level (Fig 8). The more error-prone analysis at bacterial cell level, which relies on proper segmentation of single cells, showed no clear difference between DSM43989 pEPR1-p45*gfp* compared to ATCC 13032 pEPR1-p45gfp while the remaining two *C*. *diphtheriae* strains showed a delayed co-localization with acidic compartments of J774E macrophages (Fig 8A). In contrast, all *C*. *diphtheriae* strains showed a delay of phagolysosome formation compared to *C*. *glutamicum* when images of THP-1 cells were analyzed (Fig 8A). Automated analyses of fluorescence microscopy images using the more robust, pixel-based method showed consistently that all GFP-tagged *C*. *diphtheriae* strains were able to slow down phagosome maturation in murine and human cell lines compared to the non-pathogenic GFP-expressing *C*. *glutamicum* (Fig 8B).

### Response of macrophage cell lines to infection

**Fig 9:**
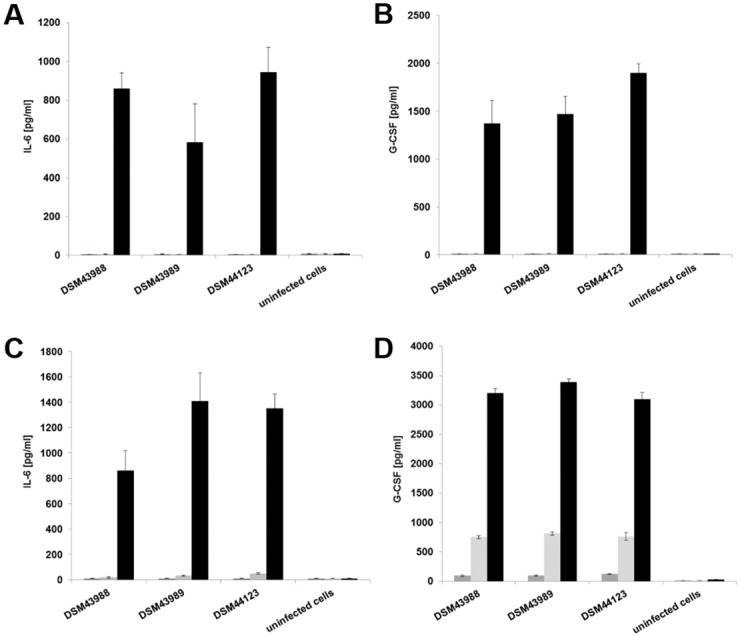
Cytokine ELISA of macrophages after infection with *C*. *diphtheriae*. Supernatants of J774E (A, B) and THP-1 (C, D) cells infected with *C*. *diphtheriae* were collected at 2 (black bars), 8 (grey bars) and 20 h (light grey bars) post-infection and used as samples for determination of (A, C) IL-6 and (B, D) G-CSF concentrations. Data shown are mean values of three independent biological replicates each performed in triplicates ± standard deviation.

In addition to phagolysosome formation, macrophage function was addressed by monitoring different signaling pathways. First, supernatants of J774E and THP-1 cells infected with *C*. *diphtheriae* strains at an MOI of 10 for 2, 8 and 20 hours were collected and used for determination of IL-6 and G-CSF secretion. In these measurements, no statistically relevant differences in the behavior of strain DSM43989 were observed. After 20 h, all strains reached about 800 pg ml^-1^ of IL-6 and 1,500 pg ml^-1^ for G-CSF in J774E supernatants (Fig 9A and 9B). Cytokine secretion was stronger in THP-1 cells reaching about 1,400 pg ml^-1^ for IL-6 (with exception of DSM439888, which reached only about 900 pg ml^-1^) and 3,000 pg ml^-1^ for G-CSF (Fig 9C and 9D).

**Fig 10:**
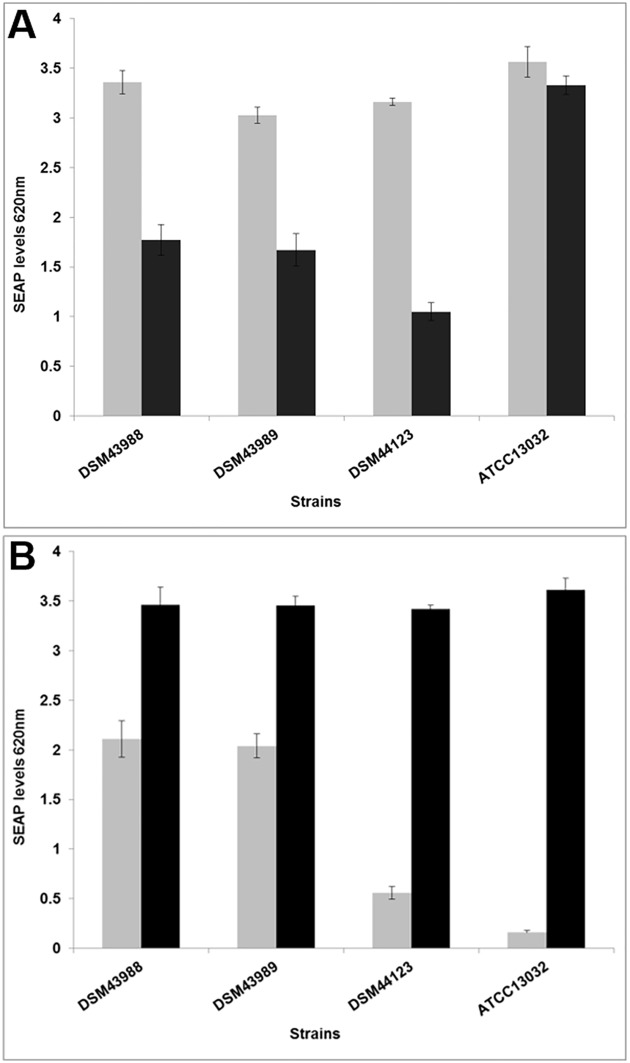
NF-κB activation in THP1-Blue NF-κB reporter cells after *C*. *diphtheriae* infection. THP1-Blue NF-κB cells were incubated for 20 h with A) viable and B) UV-killed bacteria of the non-pathogenic *C*. *glutamicum* ATCC13032 and pathogenic *C*. *diphtheriae* strains DSM43988, DSM43989 and DSM44123 at an MOI of 1 (grey bars), 10 (black bars). Supernatants were taken and mixed with QuantiBlue SEAP detection solution leading to a change in color upon NF-κB activation. Data shown are mean values of three independent biological replicates each performed in triplicates ± standard deviation.

As a second approach, NF-κB induction was analyzed in response to infection with *C*. *diphtheriae*. Cells of the reporter cell line THP-1-Blue NF-κB were incubated for 20 hours with viable and UV-killed bacteria of non-pathogenic *C*. *glutamicum* ATCC 13032 and pathogenic *C*. *diphtheriae* DSM43988, DSM43989 and DSM44123 (Fig 10). Viable *C*. *diphtheriae* led to strongest NF-κB activation when MOI 1 was tested, while the MOI of 10 led to decreased NF-κB induction, which might be due to detrimental effects on the cells. The activation by the non-pathogenic *C*. *glutamicum* was independent of its MOI (Fig 10A). In contrast, a weaker NF-κB activation was observed for MOI 1 compared to MOI 10, when dead bacteria were used (Fig 10B). Therefore, the activation by dead bacteria seems to be dose-dependent. Moreover, dead bacteria reached the values obtained for viable cells only in case 10-fold higher MOIs were applied.

Taken together, these data indicate a functional NF-κB signal transduction pathway in THP-1 cells, indicating that delay of phagolysosome formation is caused by a specific mechanism, which is independent of *C*. *diphtheriae* mycolates.

### Comparative genome sequence analysis

Since we were interested in the molecular background of mycolic acid deficiency in DSM43989, the genome sequence of this strain was determined together with DSM44123. The genomes of strains DSM43989 and DSM44123 were assembled into 45 and 28 contigs with total assembly sizes of 2.45 Mb and 2.37 Mb, respectively. The genome sequences of these strains are available from the GenBank with the accession numbers LJXS00000000 and LJXR00000000, respectively. The genome sequence of strain DSM43988 was obtained from the GenBank and was included in the comparative analyses (Accession No. AUZN00000000, [2]).

Strain DSM43989 is distinct from DSM44123 and DSM43988 as the former belong to sequence type (ST) 44 and both the latter strains are ST26. A closer bioinformatics analysis revealed that all genes necessary for mycolic acid synthesis in corynebacteria were present in genomes of the studied *C*. *diphtheriae* strains including DSM43989 (Table 3). Since *C*. *glutamicum* strains carrying deletions of *otsA* and *treY* or of *otsA*, *treY* and *treS* were also devoid of trehalosyl mycolates [41,50,51], the genome sequences of these *C*. *diphtheriae* strains were also BLAST-searched for corresponding genes. While the *treYZ* pathway was absent in all *C*. *diphtheriae* genome sequences, *otsAB*, different mycolyltransferase-encoding genes and genes coding for enzymes involved in glycogen metabolism were observed in all three genomes.

**Table 3:** Presence and absence of genes involved in mycolic acid synthesis in *C*. *diphtheriae* strains. The NCTC 13129 sequence was used as comparison. DIP1118, which is absent in DSM43988 and DSM43989 is annotated as two smaller genes in DSM44123. Partial genes, potentially due to gaps in the genomic sequence, are shaded in grey. DIP0789, highlighted in yellow, is a pseudogene in DSM43989.

NCTC 13129	Gene ID	Function	DSM43988	DSM43989	DSM44123
DIP0658	*accD1/pccB1*	Propionyl CoA carboxylase beta chain 1	B178_02716	AO271_00190	AOT42_01525
DIP0660	*pccB2*	Propionyl CoA carboxylase beta chain 2	B178_02731	AO271_00180	AOT42_01535
DIP0740	*accD2*	Acyl-CoA carboxylase beta subunit	B178_03126	AO271_10895	AOT42_01905
DIP0649	*accBC*	Acyl-CoA carboxylase alpha subunit	B178_03136	AO271_00235	AOT42_01480
DIP0787	*accDA*	Acetyl-CoA carboxylase carboxyl transferase subunit	B178_03318—B178_03323	AO271_01375	AOT42_07885
DIP0657	*accE*	Acetyl-CoA carboxylase subunit	B178_02711	AO271_00195	AOT42_01520
DIP2188	-	Propionyl CoA carboxylase beta	B178_09863	AO271_04260	AO271_04260
DIP2183	*mmpL1*	Putative drug exporter of the RND superfamily	B178_09823	AO271_04230	AOT42_05820
DIP0250	*mmpL2*	Putative drug exporter of the RND superfamily	B178_00902—B178_00917	AO271_09785	AOT42_10445
DIP1118	-	Integral membrane protein (MmpL family)	-	AOT42_03000-AOT42_03005	-
DIP1812	*cmrA*	*Corynebacterineae* mycolate reductase A	B178_08010	AO271_08895	AOT42_06590
DIP0365	*cmtA/slpA*	Trehalose corynomycolyl transferase A (surface-layer protein A)	B178_01563	AO271_04745	AOT42_00045
DIP2194	*cmtB*	Trehalose corynomycolyl transferase B	B178_09908	AO271_04295	AOT42_05760
DIP2193	*cmtC/csp1*	Trehalose corynomycolyl transferase C	B178_09898	AO271_04285	AOT42_05770
DIP2339	*cmtD*	Trehalose corynomycolyl transferase D	B178_10633	AO271_06825	AOT42_03595
DIP1966	*otsA*	Alpha,alpha-trehalose-phosphate synthase	B178_08689	AO271_05880	AOT42_07065
DIP1968	*otsB*	Trehalose 6-phosphate phosphatase	B178_08699	AO271_05870	AOT42_07055
DIP1066	*glgE*	Alpha-1,4-glucan:maltose-1-phosphate maltosyltransferase	B178_04681	AO271_03420	AOT42_02730
DIP1065	*glgB*	1,4-alpha-glucan (glycogen) branching enzyme	B178_04676	AO271_03415	AOT42_02725
DIP1572	*glgX*	Glycogen debranching protein	B178_06829	AO271_08725	AOT42_09590
DIP1846	*fas*	Fatty acid synthase	B178_08075-B178_08090	AO271_10830	AOT42_06660
DIP1116	-	Putative exported esterase/hydrolase	-	AO271_03670	AOT42_02980
DIP1200	-	Putative membrane protein	B178_05156	AO271_06340	AOT42_08635
DIP2015	-	Putative exported lipase	-	-	-
DIP1472	-	4'-Phosphopentethenyl transferase	B178_06664	AO271_07215	AOT42_04865
DIP1845	-	4'-Phosphopentethenyl transferase	B178_08070	AO271_10835	AOT42_06655
DIP2191	*elrF*	Envelope lipids regulation factor	B178_09888	AO271_04275	AOT42_05780
DIP2189	*pks13*	Polyketide synthase involved in mycolic acid biosynthesis	B178_09868	AO271_04265	AOT42_05790
DIP2190	*fadD1*	Acyl-CoA synthetase	B178_09883	AO271_04270	AOT42_05785
DIP1725	*fadD2*	Acyl-CoA synthetase	B178_07560	AO271_02620	AOT42_06170
DIP0358	*fadD3*	Acyl-CoA synthetase	B178_01528	AO271_04780	AOT42_00010
DIP1038	*fadD4*	Acyl-CoA synthetase	B178_04551	AO271_03280	AOT42_02595
DIP0386	*fadD6*	Acyl-CoA synthetase	B178_01673	AO271_04655	AOT42_00130
DIP0387	*fadD7*	Acyl-CoA synthetase	B178_01678	AO271_04660	AOT42_00135
DIP0789	*echA2*	Enoyl-CoA hydratase	B178_03333	AO271_01385	AOT42_07875
DIP0885	*echA5*	3-hydroxyisobutyryl-CoA hydrolase	B178_03750	AO271_01750	AOT42_07510
DIP0421	*menB*	1,4-dihydroxy-2-naphthoyl-CoA synthase (DHNA-CoA synthase)	B178_01848	AO271_04490	AOT42_00305

A BLAST search of genes identified in strain NCTC 13129 with a potential involvement in the mycolic acid biosynthesis revealed their presence in all three *C*. *diphtheriae* genomes (Table 3). However, *DIP0789*, annotated to encode an enoyl-CoA hydratase, was a pseudogene in DSM43989.

### The role of the putative enoyl-CoA hydratase

To investigate the putative role of the DIP0789, the corresponding gene *B178_03333* was amplified from chromosomal DNA of DSM43988 and cloned into expression plasmid pZ8-1. When complementation of the growth defect was tested, no positive effect of the corresponding plasmid was observed: strain DSM43989 transformed with the empty vector control pZ8-1 reached a doubling time of 131 ± 22 min, while the overexpression of *B178_03333* in DSM43989 led to a doubling time of 132 ± 19 min (means and standard deviations from three independent experiments). Since the synthesis of the mycolic layer is a highly complex process not only the presence of a protein might be important, but also time and activity level might be crucial for full complementation of a defect. Therefore, as a more sensitive approach, cell wall extracts were investigated by thin-layer chromatography. In fact, in contrast to the empty vector control, the complementation of DSM43989 with *B178_03333* clearly restored the CMAMES levels in the DSM43989 strain carrying the corresponding overexpression vector and as well as the production of TDM (Fig 2A and 2B), showing that the role of the enoyl-CoA hydratase is key in restoring the function. According to the literature, the MS analysis of CMAMES from *C*. *diphtheriae* and other members of the genus should reveal peaks within the 500–595 m/z range that account for the presence of the most abundant species, i.e. C30-C34 [52,53]. MS data revealed the total absence of peaks within that m/z range in the DSM43989 and DSM43989 empty vector strains for both cell wall bound lipids and total lipids methyl ester fractions. These peaks were restored by complementation with *B178_03333*, although the heterogeneity and the abundance of cell wall bound corynomycolic acids methyl esters could not be entirely restored (S1 Fig). The results obtained suggest that the putative enoyl-CoA hydratase DIP0789 is involved in mycolic acid synthesis in *C*. *diphtheriae*.

## Discussion

Almost all *Corynebacterinae* are characterized by a typical, lipid-rich cell wall structure with an outer membrane layer dominated by mycolic or corynomycolic acids linked to arabinogalactan or esterified to trehalose as trehalose monomycolate (TMM) and trehalose dimycolate (TDM) [54]. Due to the hydrophobic character of the mycolic acid layer, it functions as a permeability barrier [55,56], and might prevent the access of harmful substances. In line with this idea, the mycolate-free strain DSM43989 showed in fact a higher susceptibility to antibiotics, especially β-lactam antibiotics.

Besides the passive function as building block for a permeability barrier, TDMs of pathogenic mycobacteria have been recognized for decades for their role in induction of inflammatory responses, granuloma formation and adjuvant activity [57,58]. While mycolic acid synthesis is essential in *M*. *tuberculosis*, *C*. *diphtheriae* is not only viable without mycolic acids, but also survival in macrophages seems not to be impaired. This result could not be observed for the non-pathogenic strain *C*. *glutamicum* [17]. This is astonishing on first sight, since in analogy to mycobacteria a function of corynotrehalosylmycolates in pathogenicity was at least discussed [6]. However, at least two mycolic acid-free pathogenic *Corynebacterium* species are known, *Corynebacterium amycolatum* and *Corynebacterium kroppenstedtii* [59–62], which successfully interact with human hosts, showing at mycolic acids may not be crucial in *Corynebacterium* host interaction. In fact, for *C*. *diphtheriae* pathogenicity determinants like DIP0733 were described recently [22,63], which might comprise similar functions as trehalosyldimycolates of *M*. *tuberculosis*.

Interestingly, Park-Williams strain DSM43989 was found to be mycolic acid deficient in this study, despite the fact that all genes previously known to be necessary for mycolic acid synthesis were found intact. Comparative genome sequence analyses revealed the DIP0789 as a pseudogene in DSM43989 and partial restoration of corynomycolic acids following complementation with a functional copy of the gene from DSM43988 revealed a previously unknown role in corynomycolate biosynthesis. Interestingly, no full complementation was achieved. This observation might either hint to a complex regulation and activity of the corresponding gene product or the existence of additional unknown factors responsible for the lack of mycolates in DSM43989. However, partial complementation of corynomycolic acid biosynthesis in corynomycolate-deficient mutants is not without precedent; corynomycolic acid biosynthesis was only partially restored in a *C*. *glutamicum pks13* mutant following introduction of a *pks13* containing plasmid [64].

DIP0789 is annotated as an enoyl-CoA hydratase, an enzyme known to be involved in the isomerization of 2-trans-enoyl-ACP to 3-cis-enoyl-ACP in FAS-II fatty acid biosynthetic pathway (hydratase/isomerase superfamily: Pfam000378; [11,65]). The gene is present in single copy in most *C*. *diphtheriae* genomes but it is a pseudogene in strains DSM43989 and NCTC 05011 [24,66]; genome accession no: AJVH00000000.1). It is not clear if strain NCTC 05011 also expressed the mycolic acid deficiency phenotype. This gene has been annotated as two smaller genes in strain PW8 (*CDPW8_0784* and *CDPW8_0785*; genome accession no: CP003216.1) but it may just be a miss annotation as the nucleotide sequence of DIP0789 show 100% coverage and 99% identity in PW8.

A protein BLAST search revealed a wide presence of homologous genes within CMNR group. While corynebacterial species including *C*. *ulcerans*, *C*. *pseudotuberculosis* and *C*. *glutamicum* appears to possess a single homolog of enoyl-CoA hydratase encoding gene, multiple copies have been observed in some members of genera *Mycobacterium* and *Rhodococcus*. Surprisingly, many paralogues of *echA* in *M*. *tuberculosis* appear to be missing canonical active site residues essential for isomerase function [67]. Interestingly, EchA6 from *M*. *tuberculosis* appears to be a non-catalytic protein that plays an important role in mycolic acid biosynthesis, possibly as functioning as a conduit for long chain fatty acids recycled into the mycolate biosynthesis machinery.

Enoyl-CoA hydratase enzymes had been reported in the context of the catabolism of fatty acids [68,69], but the non-catalytic EchA6 encode by Rv0905 has recently been described as a fatty acid shuttle in mycobacteria, where it binds to acyl-CoA units, interacts with FAS-II module components and its depletion leads to the suppression of mycolic acid biosynthesis [67]. Although it was reported as essential under the tested conditions, mutant strains were obtained in previous screenings [70,71]. The functional link between EchA6 and DIP0789 is not necessarily obvious, and since there is only one FAS-I system in *C*. *diphtheriae* and the *Corynebacterium* genus lacks of a FAS-II system [43,72], clarification whether Rv0905 and DIP0789 have the same role or not is needed. A BLAST analysis reveals a 38% of identity for their protein sequences, but more interesting, both genes are in the same cluster with *accD* genes Rv0904c and DIP0787, respectively, showing 49% of identity between their protein sequences. In *C*. *glutamicum*, *accD2* and *accD3* genes have been found to be involved in mycolic acids biosynthesis [29], but the synteny between the enoyl-CoA hydratase and the AccD is missing. Nevertheless, in *C*. *diphtheriae*, it seems plausible that the Rv0905 homolog could have a role in mycolic acids biosynthesis by carrying acyl-CoA units between β-oxidation and lipid biosynthesis, connecting both pathways in a regulatory fashion.

In summary, the results obtained in this study show that mycolic acids may have varying functions in different members of the CMNR group. Furthermore, our data indicate that also the complex synthesis pathway of the cell envelope including the corynomycolic acid layer is not fully understood in corynebacteria.

## Supporting information

S1 Fig Mass spectrometry analyses of lipid extracts from different *Corynebacterium* strains. Analyses reveal the total absence of peaks within the 500–595 m/z range (indicated by black line under abscissa) accounting for the most abundant species C30-C34 in DSM43989 and DSM43989 pZ8-1 for both cell wall bound (A) and total lipid methyl ester fractions (B). Peaks were restored under the overexpression of B178_03333 in DSM43989 and were also present in DSM43988 and DSM44123 as well as in *C*. *diphtheriae* strain INCA402 and *C*. *glutamicum* wild type ATCC 13032 used as additional controls.
(TIF)
