# Supplementary figures and images for: Analysis of Corynebacterium diphtheriae macrophage interaction: Dispensability of corynomycolic acids for inhibition of phagolysosome maturation and identification of a new gene involved in synthesis of the corynomycolic acid layer
Source: PLoS One. 2017 Jul 7;12(7):e0180105. doi: 10.1371/journal.pone.0180105 (PMC5501465; doi:10.1371/journal.pone.0180105)

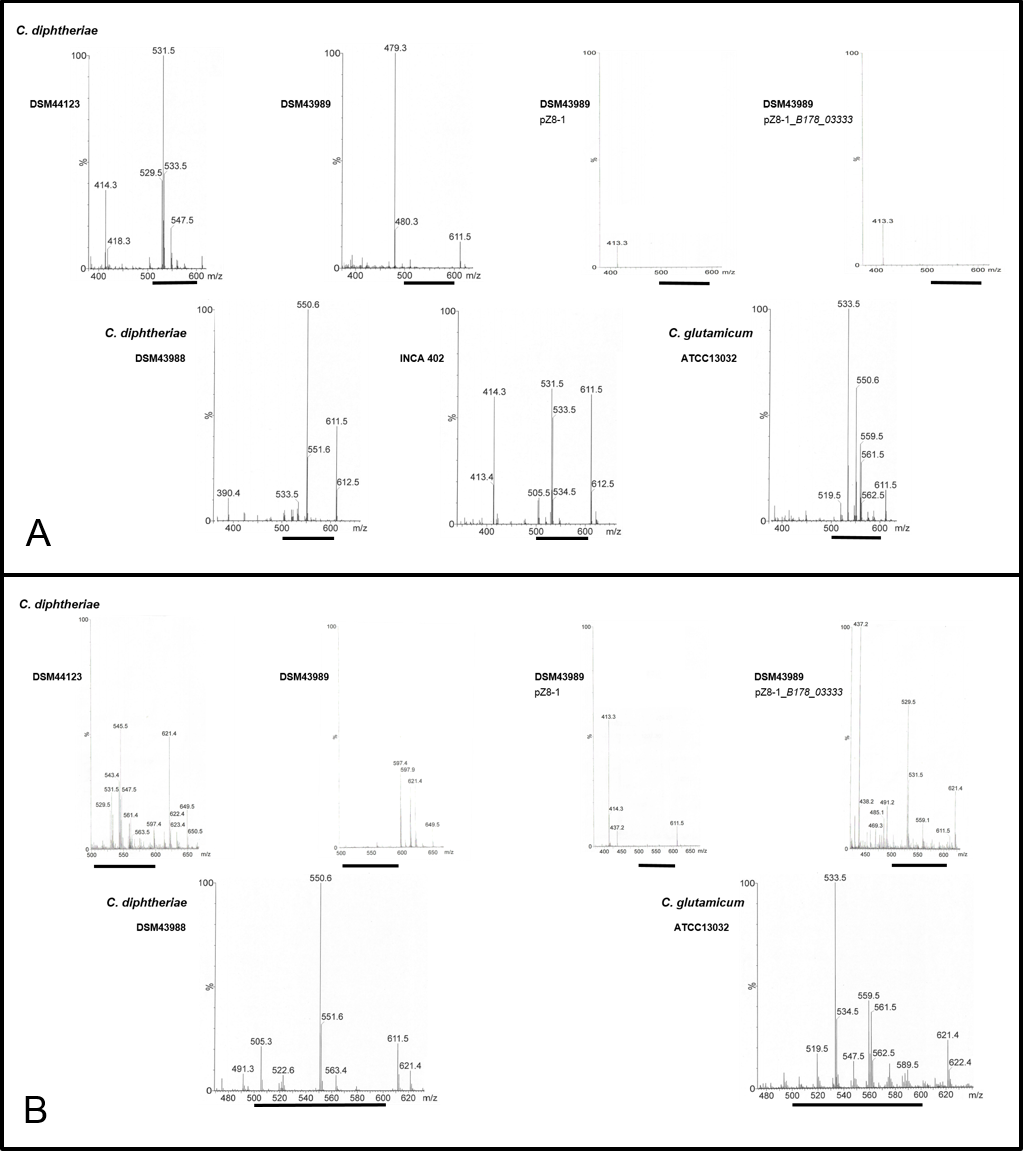

Supplement: S1 Fig — Analyses reveal the total absence of peaks within the 500–595 m/z range (indicated by black line under abscissa) accounting for the most abundant species C30-C34 in DSM43989 and DSM43989 pZ8-1 for both cell wall bound (A) and total lipid methyl ester fractions (B). Peaks were restored under the overexpression of B178_03333 in DSM43989 and were also present in DSM43988 and DSM44123 as well as in C. diphtheriae strain INCA402 and C. glutamicum wild type ATCC 13032 used as additional controls. (TIF) [file pone.0180105.s001.tif]
